# Characterization of the roles of activated charcoal and Chelex in the induction of PrfA regulon expression in complex medium

**DOI:** 10.1371/journal.pone.0250989

**Published:** 2021-04-29

**Authors:** Ahmed Gaballa, Sriya Sunil, Etienne Doll, Sarah I. Murphy, Tyler Bechtel, Veronica Guariglia-Oropeza, Martin Wiedmann

**Affiliations:** Department of Food Science, Cornell University, Ithaca, New York, United States of America; Western Michigan University, UNITED STATES

## Abstract

The foodborne pathogen *Listeria monocytogenes* is able to survive across a wide range of intra- and extra-host environments by appropriately modulating gene expression patterns in response to different stimuli. Positive Regulatory Factor A (PrfA) is the major transcriptional regulator of virulence gene expression in *L*. *monocytogenes*. It has long been known that activated charcoal is required to induce the expression of PrfA-regulated genes in complex media, such as Brain Heart Infusion (BHI), but not in chemically defined media. In this study, we show that the expression of the PrfA-regulated *hly*, which encodes listeriolysin O, is induced 5- and 8-fold in *L*. *monocytogenes* cells grown in Chelex-treated BHI (Ch-BHI) and in the presence of activated charcoal (AC-BHI), respectively, relative to cells grown in BHI medium. Specifically, we show that metal ions present in BHI broth plays a role in the reduced expression of the PrfA regulon. In addition, we show that expression of *hly* is induced when the levels of bioavailable extra- or intercellular iron are reduced. *L*. *monocytogenes* cells grown Ch-BHI and AC-BHI media showed similar levels of resistance to the iron-activated antibiotic, streptonigrin, indicating that activated charcoal reduces the intracellular labile iron pool. Metal depletion and exogenously added glutathione contributed synergistically to PrfA-regulated gene expression since glutathione further increased *hly* expression in metal-depleted BHI but not in BHI medium. Analyses of transcriptional reporter fusion expression patterns revealed that genes in the PrfA regulon are differentially expressed in response to metal depletion, metal excess and exogenous glutathione. Our results suggest that metal ion abundance plays a role in modulating expression of PrfA-regulated virulence genes in *L*. *monocytogenes*.

## Introduction

*Listeria monocytogenes* is a Gram-positive foodborne pathogen that can survive across a wide range of extra- and intra-host environments [1]. In a mammalian host, *L*. *monocytogenes* can cause listeriosis [2], a disease with high mortality, likely related to the fact that this pathogen has the ability to invade multiple tissue and cell types. Additionally, in an extra-host environment, *L*. *monocytogenes* can survive in the presence of multiple bacterial growth deterrents such as low temperature, low pH, variable nutrient availabilities, and the presence of some anti-microbial compounds [3,4]. *L*. *monocytogenes’* ability to survive under a wide-range of environmental conditions is supported by coordinated expression of elaborate networks of genes that are involved in virulence and stress response [5–8]. The majority of genes involved in virulence and stress response are regulated, respectively, by the transcriptional activator Positive Regulatory Factor A (PrfA) and the alternative sigma factor σ^B^ [5,9–12].

PrfA is a dimeric protein of the cAMP receptor protein (Crp)-fumarate reductase regulator (Fnr) superfamily [13]. Members of the Crp/Fnr family activate transcription by binding to a conserved DNA motif located upstream and sometimes overlapping a promoter’s -35 region [14]. PrfA activates transcription by binding to an inverted repeat DNA motif called the PrfA-box, which overlaps the -35 promoter region of multiple virulence-related genes in *L*. *monocytogenes* [15,16]. Regulators in the Crp-family are activated when an allosteric co-activator, generally a small molecule, binds to the regulator’s N-terminal region, causing conformational changes that allow the Helix-Turn-Helix region in the protein’s C-terminal domain to bind DNA [17]. Recently, glutathione (GSH) was identified as an allosteric activator of PrfA [18].

In addition to its activation by GSH, PrfA also is regulated at transcriptional, post-transcriptional and post-translational levels (for recent reviews see [6,7]). At the transcriptional level, *prfA* is transcribed from 3 different σ^A^ and/or σ^B^-dependent promoters [19–22]. At the post- transcriptional level, PrfA translation is modulated by a thermo-switch, while carbon source, redox status, branched-chain amino acids and oligopeptides all appear to modulate PrfA activity post-translationally [23–26]. PrfA’s multifaceted regulation ensures appropriate spatial and temporal expression of different virulence-related genes during the infection process [7].

PrfA regulates transcription of a small set of genes as well as one non-coding RNA; all PrfA-dependent genes appear to be involved in virulence-related functions (for review, see [3,16]). Among these genes are *hly*, *plcA*, *plcB* and *mpl*, which encode a cholesterol-activated pore forming toxin listeriolysin O (LLO), a phosphatidylinositol phospholipase, a lecithinase and a metal-dependent protease, respectively; all are required for *L*. *monocytogenes* escape from primary and/or secondary vacuoles. Subsequently, at a later stage of infection, PrfA activates expression of *actA*, which encodes the actin-recruiting protein required for host cell actin polymerization-based bacterial movement and cell-to-cell spread, and of *hpt*, which encodes the glucose-6-phosphate uptake system required for growth in the host cytosol [27–29].

**Fig 1:**
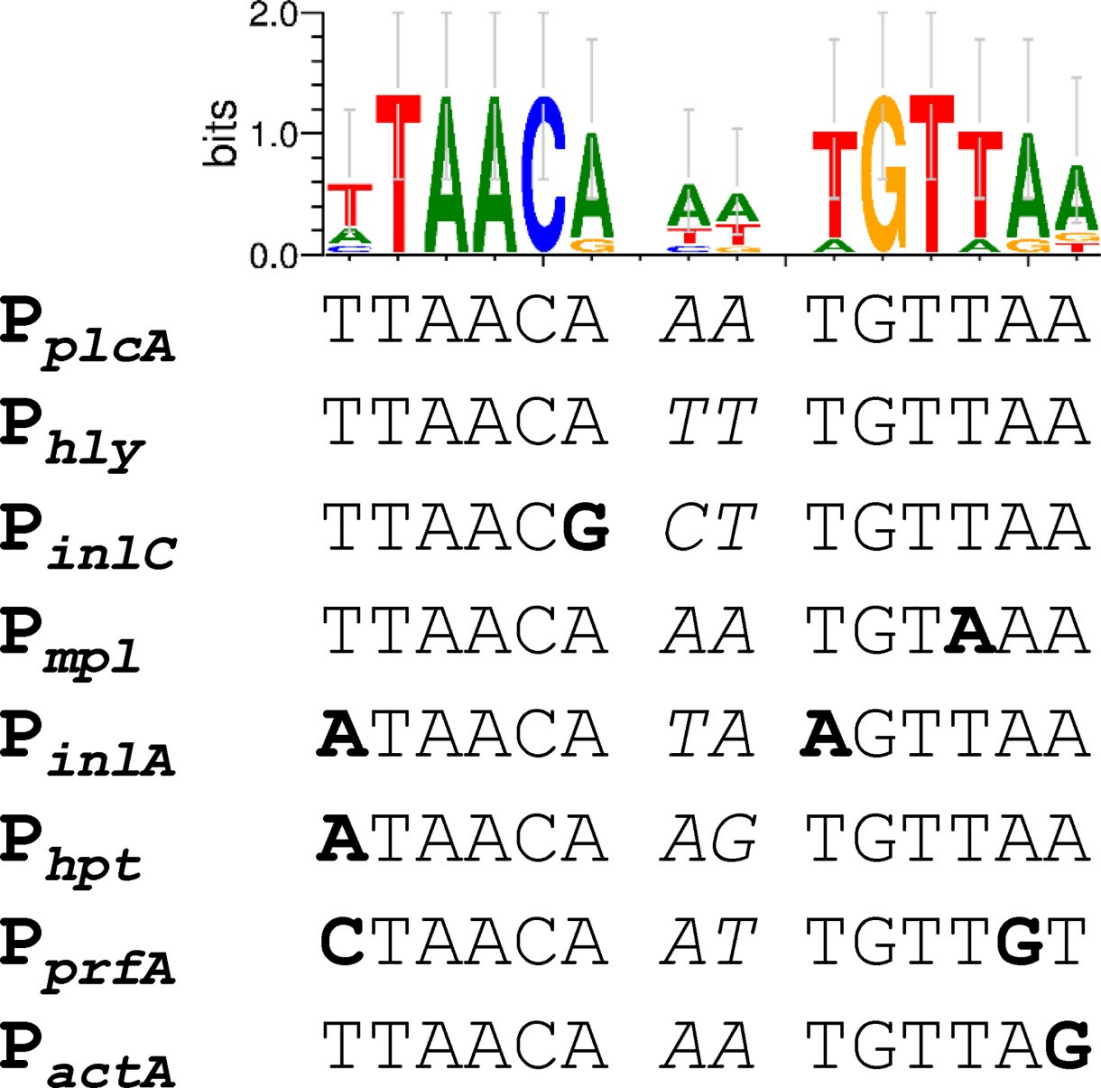
Conservation of the PrfA box sequences in the promoter region of PrfA-regulated genes. The PrfA box sequence WebLogo is presented above a multiple sequence alignment of known PrfA boxes extracted from *L*. *monocytogenes* strain 10403S whole genome sequence data deposited in NCBI under the accession number NC_017544.1. Bold letters indicate nucleotides that deviate from providing a canonical PrfA box hairpin. Italicized letters represent non-conserved nucleotides in the spacer region.

Genes in the PrfA regulon are differentially expressed through modulation of PrfA levels and activity. As PrfA binding to the PrfA box is a prerequisite for inducing transcription of PrfA-regulated genes (Fig 1, [16]), relative conservation of the PrfA-box has been examined as one mechanism that guides differential PrfA regulon expression [6,7,30,31]. For example, transcription of genes with a canonical PrfA box, such as *hly* and *plcA*, requires lower levels of active PrfA relative to transcription of genes with PrfA boxes that deviate from the consensus sequence, such as *actA* and *plcB* (Fig 1) [28,32,33]. Hence, measurement of *hly* and *actA* expression levels has been used as a proxy for “relaxed” and “stringent” requirements, respectively, for PrfA levels and activities [34].

It has been widely noted that *L*. *monocytogenes* PrfA-regulated genes are expressed at lower levels in complex media such as BHI or LB broth than in chemically defined media [26,32]. Activated charcoal and Amberlite XAD are commonly used to induce the expression of PrfA-regulated genes in complex growth media [27,35]. Recently, it has been shown that Amberlite XAD induces expression of the PrfA regulon in BHI by depleting oligopeptides, which either inhibit or activate PrfA depending on their cysteine content [23]. On the other hand, the specific mechanism by which charcoal activates PrfA-dependent gene expression in BHI remains unknown [36–38].

In this work, we show that metal abundance in BHI medium negatively influences the expression of PrfA-regulated genes. Moreover, we show that exogenous GSH induces expression of the PrfA-regulated *hly* gene in metal-depleted BHI, but not in BHI medium. This data suggests that the relative abundance of metal ion may modulate virulence gene expression in *L*. *monocytogenes*.

## Materials and methods

### Bacterial strains and media

Bacterial strains and cloning vectors used in this study are listed in S1 Table. Brain Heart Infusion (BHI, Becton Dickinson [BD], Franklin Lakes, NJ cat. # 237200) and Difco Lennox broth (LB; BD cat. # 240230) were prepared according to the manufacturers’ recommendations and used for growth of *L*. *monocytogenes* and *E*. *coli*, respectively. To prepare BHI broth with activated charcoal (AC-BHI), 0.2% w/v of activated charcoal (Sigma, cat. # C9157) was added to BHI broth before autoclaving at 121°C for 15 min. Chelex-treated BHI (Ch-BHI) was prepared by adding 5% w/v Chelex-100 (BioRad cat. # 1422832) to BHI broth, with subsequent slow stirring for 30 min. Chelex resin was removed by filtration using paper filter (Whatmann 1, 110mm ∅Cat. # 1001 110) and the treated medium was then filter sterilized using a filtration system with 0.22μm cellulose acetate filter (Corning Cat. # 430767). Magnesium chloride and manganese chloride (1mM and 1μM, respectively) were added aseptically to the Ch-BHI medium [39]. Disposable plastic vessels were used in preparation of metal-depleted medium and in bacterial cultures to avoid any metal contamination from re-usable glassware [40]. Iron, zinc, GSH, hemin, dipyridyl (DP) or ethylenediamine-N,N′-bis(2-hydroxyphenylacetic acid) (EDDHA) were added aseptically from filter-sterilized stock solutions to the media directly prior to bacterial inoculation. Solid media were prepared by adding 15g/L of bacteriological agar (Difco, BD) before autoclaving.

### Construction of GFP transcriptional reporter fusions

The codon-optimized eGFP coding region was amplified from pKSV7-P_*lmo2230*_::*egfp* [41] and the promoter regions from each *hly*, *actA*, *mpl*, *hpt* and *inlA* were amplified from *L*. *monocytogenes* 10403S genomic DNA using primers listed in S2 Table and using Q5 high-fidelity DNA Polymerase (NEB) according to the manufacturer’s recommendations. PCR products were purified and the eGFP coding region was cloned downstream of each promoter regions into the pPL2 vector using three-way ligation [42]. pPL2 [43] is an integrative vector that encodes a PSA integrase and integrates at the *attPP′* attachment site, which is located at the 3′ end of the arginine tRNA gene in *L*. *monocytogenes*. To ensure expression comparability among the constructs, all constructs were designed to have an identical ribosome binding site (RBS; UAGGAGG) 7 bases upstream of the *egfp* start codon [44]. The constructs were confirmed by DNA sequencing and introduced into *L*. *monocytogenes* by conjugation [45].

### GFP quantification

GFP fluorescence levels were measured in *L*. *monocytogenes* clones bearing different promoter-eGFP transcription fusions. Pre-cultures were prepared by inoculating a single colony of the appropriate strain into 5ml of BHI supplemented to a final concentration of 7.5μg/ml chloramphenicol and incubated at 37°C with shaking overnight at 200rpm (14-16h). One ml of BHI, AC-BHI or Ch-BHI in a sterile 5ml plastic tube was inoculated with 20μl of the overnight culture and incubated at 37°C with shaking to mid-exponential phase (~ 4-6h). To measure GFP fluorescence and OD_600_ in AC-BHI cultures, a brief centrifugation step at 110 g for 3 min was implemented to remove activated charcoal particles [46], which also resulted in a nominal loss of bacterial cells. In addition, tubes with un-inoculated AC-BHI were incubated, processed in the same manner, and used as blanks for OD measurements; OD_600_ or GFP fluorescence measurements for these blanks showed minimal differences to a PBS control. Cultures were transferred to 96-deep well plates (Thermo Cat. # AB-0765) and cells were collected by centrifugation at 3200 g for 10 min. Supernatants were discarded; cells were washed twice in sterile PBS and diluted to obtain 100μl of a suspension with an OD_600_ of 0.2–0.6 in 96-well plates with black walls and clear bottoms (VWR Cat. # 655097). Optical densities were measured at 600nm and fluorescence readings were measured at 485nm (excitation) and 528nm (emission). GFP fluorescence levels were normalized by the culture OD_600_. While some samples were lost during the washing step, resulting in different numbers of replicates among some conditions, at least 3 independent biological replicates were available for all statistical analyses, except for AC-BHI cultures for which at least 6 independent biological replicates were available for statistical analyses. When needed, unequal sample size was taken into account in the statistical analyses.

### Streptonigrin susceptibility assay

Single colonies of *L*. *monocytogenes* were inoculated into 5 ml BHI, followed by incubation overnight (14-16h) at 37°C with shaking at 200 rpm. Five ml BHI were inoculated from the pre-culture at 1/200 dilution, followed by incubation at 37°C with shaking at 200rpm until cultures reach an OD_600_ of 0.4 to 0.6. Four ml of melted BHI, AC-BHI or Ch-BHI soft agar media were mixed with 100μl of culture and poured onto Petri plates with 15 ml of the corresponding solid medium; plates were dried for 5 min. Paper disks were impregnated with 15 or 25 μg of streptonigrin and laid on top of the soft agar. Plates were incubated for 14–16 h at 37°C and the zone of inhibition around the disk was measured for 6 independent biological replicates.

### Statistical analyses

Statistical analyses were done using GraphPad Prism-8 and R version 3.6.0 [47] using RStudio (version 1.2.1335, RStudio Inc., Boston, MA). Statistical analyses were done using the absolute measurement values (e.g., GFP fluorescence level normalized by culture OD_600_) from at least three independent biological replicates that each started from different bacterial colony. Data were analyzed for multiple comparisons using ANOVA tests with the appropriate post hoc comparisons. To determine whether values (e.g., P_*hly*_-eGFP fluorescence/OD_600_) for specific growth conditions differed from a reference condition (e.g., growth in BHI medium), a post hoc Dunnett’s test that accounts for unequal sample size was used as implemented in GraphPad Prism-8. To compare multiple growth conditions or strains, a post hoc Tukey’s test was used. Fold change to the reference treatment was calculated for graphing purpose. Data presented in the figures are the average of at least three independent biological replicates; error bars represent standard error of the mean.

## Results

### Chelex and activated charcoal activate P_*hly*_*-eGFP* expression in BHI

Previous studies that used Chelex to chelate metal ions have shown an inverse relationship between LLO production (an indictor of PrfA dependent gene expression) and iron concentration in the growth medium [35,48]. Similarliy, activated charcoal, which can bind both organic non-polar compounds as well as heavy metals [49], has also been shown to induce PrfA-dependent gene expression when present in the growth media. To further determine the mechanisms by which activated charcoal and Chelex pre-treatment induce expression of the PrfA regulon, we constructed an *L*. *monocytogenes* strain that expresses a P_*hly*_*-eGFP* transcriptional fusion. This strain was used to assess whether activated charcoal prenst in BHI (“AC-BHI”) or Chelex pre-treatment of BHI (Ch-BHI) induces the expression of P_*hly*_*-*eGFP in *L*. *monconcytgenes* grown in these media (relative to BHI that has not undergone treatments that can chelate metal ions). BHI, AC-BHI and Ch-BHI media supported the growth of *L*. *monocytogenes* cells to similar OD_600_ after 4h of incubation, suggesting that activated charcoal and Chelex did not notably affect *L*. *monocytogenes* growth in BHI. *L*. *monocytogenes* cells encoding P_*hly*_*-eGFP* and grown in Ch-BHI (10,837 ± 809 eGFP fluorescence/OD_600_) and AC-BHI (16,367 ± 450 eGFP fluorescence/OD_600_) showed 5- and 8-fold higher P_*hly*_*-*eGFP expression, respectively, relative to cells grown in BHI (2,071 ± 205 eGFP fluorescence/OD_600_). On the other hand and as expected, P_*hly*_*-*eGFP was constitutively and highly expressed in BHI in a *L*. *monocytogenes* strain that encodes a constitutively active PrfA (PrfA*), which is locked in the active form (34,161± 539.9 eGFP fluorescence/OD_600_).

### Addition of iron or zinc negates the effect of Chelex treatment on P_*hly*_*-*eGFP expression in BHI

**Fig 2:**
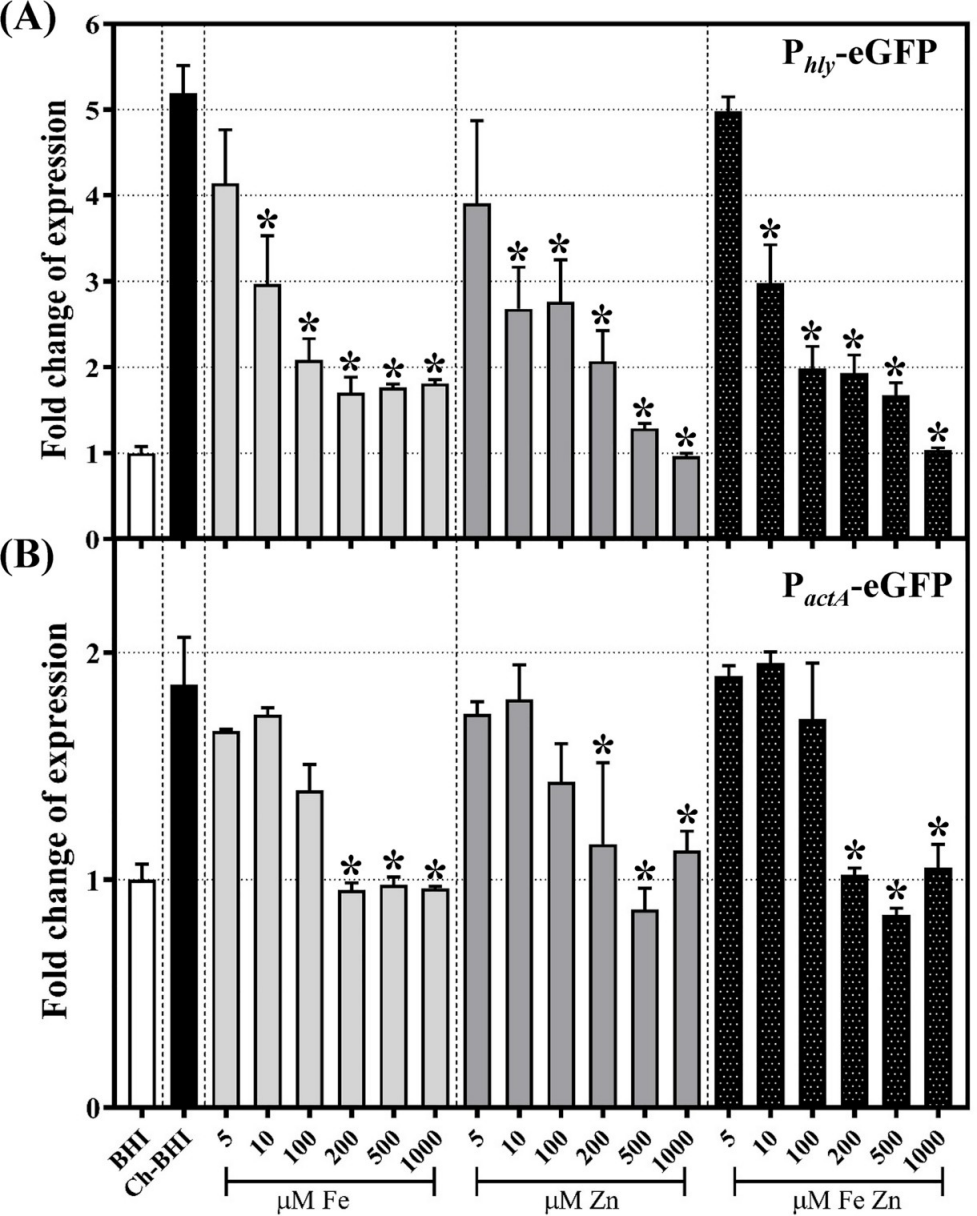
Expression of P_*hly*_-eGFP and P_*actA*_-eGFP in the presence of iron, zinc or both ion in metal-depleted BHI. *L*. *monocytogenes* strains expressing P_*hly*_-eGFP (A) or P_*actA*_-eGFP (B) reporter fusions were grown in Ch-BHI, supplemented after chelex (Ch) treatment with different concentrations of iron, zinc or both. Y axis scales are different for panels A and B. GFP fluorescence levels were measured and normalized by culture OD_600_. Fold change of expression (for graphing purpose) was calculated as a ratio of normalized GFP fluorescence level relative to the normalized fluorescence level of *L*. *monocytogenes* cells expressing the reporter fusion and growing in untreated BHI. Statistical analysis was done using the GFP/OD_600_ data from at least 3 independent biological replicates; error bars represent SEM. Asterisks denote significant difference between the normalized fluorescence level at the given condition compared to normalized fluorescence level in Ch-BHI (black bar; *p-*value *≤* 0.05,) as determined by one-way ANOVA and Dunnett’s post hoc test.

As P_*hly*_-eGFP expression was induced in cells grown in Chelex-treated BHI medium, we hypothesized that metal ion in BHI may affect PrfA-dependent expression of *hly*. Indeed, re-introduction of iron, zinc or both, to Ch-BHI at concentrations equal to or above 10μM significantly reduced P_*hly*_*-*eGFP expression by at least 2 fold relative to P_*hly*_*-*eGFP expression in Ch-BHI medium (Fig 2A). In addition, growth medium supplemented with different concentrations of iron and zinc supported *L*. *monocytogenes* growth to similar OD_600_ after 4h of incubation, suggesting that the iron and zinc, at the tested concentrations, did not affect *L*. *monocytogenes* cell growth.

To assess the effect of metal ion depletion on expression of a gene with a requirement for higher active PrfA levels, we constructed a P_*actA*_-eGFP transcriptional fusion and tested its expression in Ch-BHI. P_*actA*_*-*eGFP expression was modestly, but significantly, higher in Ch-BHI relative to expression in BHI (1.9 fold; Fig 2B; adjusted *p*-value 0.0004 as determined by one-way ANOVA and Dunnett’s post hoc test). Similar to the expression pattern for P_*hly*_*-*eGFP, P_*actA*_*-*eGFP expression in Ch-BHI was reduced by the addition of iron and zinc, albeit reduction required higher metal concentrations (i.e., ≥ 200μM) than for P_*hly*_*-*eGFP (Fig 2B).

To test if metal ions can negate the effect of activated charcoal on the expression of *hly* and *actA* in BHI medium, GFP fluorescence levels were measured in *L*. *monocytogenes* cells expressing either P_*hly*_*-*eGFP or P_*actA*_*-*eGFP and grown in AC-BHI medium supplemented with iron, zinc, or both (S1 Fig). While addition of zinc to AC-BHI medium marginally but significantly reduced the expression of P_*hly*_*-*eGFP, addition of iron or iron and zinc had no significant effect on the expression of P_*hly*_*-*eGFP (S1A Fig). Furthermore, addition of iron, zinc or both had no significant effect on P_*actA*_*-*eGFP expression in AC-BHI (S1B Fig). However, it is important to note that the activated charcoal in AC-BHI medium may continuously chelate metal ion [36,49], negating the effect of metal ion addition, which could explain the limited effect of metal ion addition on *hly* and *actA* expression found here. Pre-treatment of BHI with activated charcoal and its subsequent removal by filtration, in a similar manner to Chelex pre-treatment, was not feasible since it has been shown that pre-treatment of BHI with activated charcoal was not sufficient to induce PrfA-dependent gene expression and that activated charcoal must be present throughout the course of bacterial growth [36,37]. To rule out a possible effect of excess metal ions in the medium on GFP fluorescence per se, we measured P_*hly*_*-*eGFP levels in BHI with increasing iron and zinc concentrations. The addition of metal ions to BHI had no measurable effect on GFP fluorescence basal levels (S2 Fig). These data suggest that the presence of metal ions in BHI, specifically iron and zinc, appears to reduce the expression of the PrfA-regulated *hly* and *actA* genes.

### Chelation of intracellular Fe^2+^ or extracellular Fe^3+^ induces P_*hly*_*-*eGFP expression

**Fig 3:**
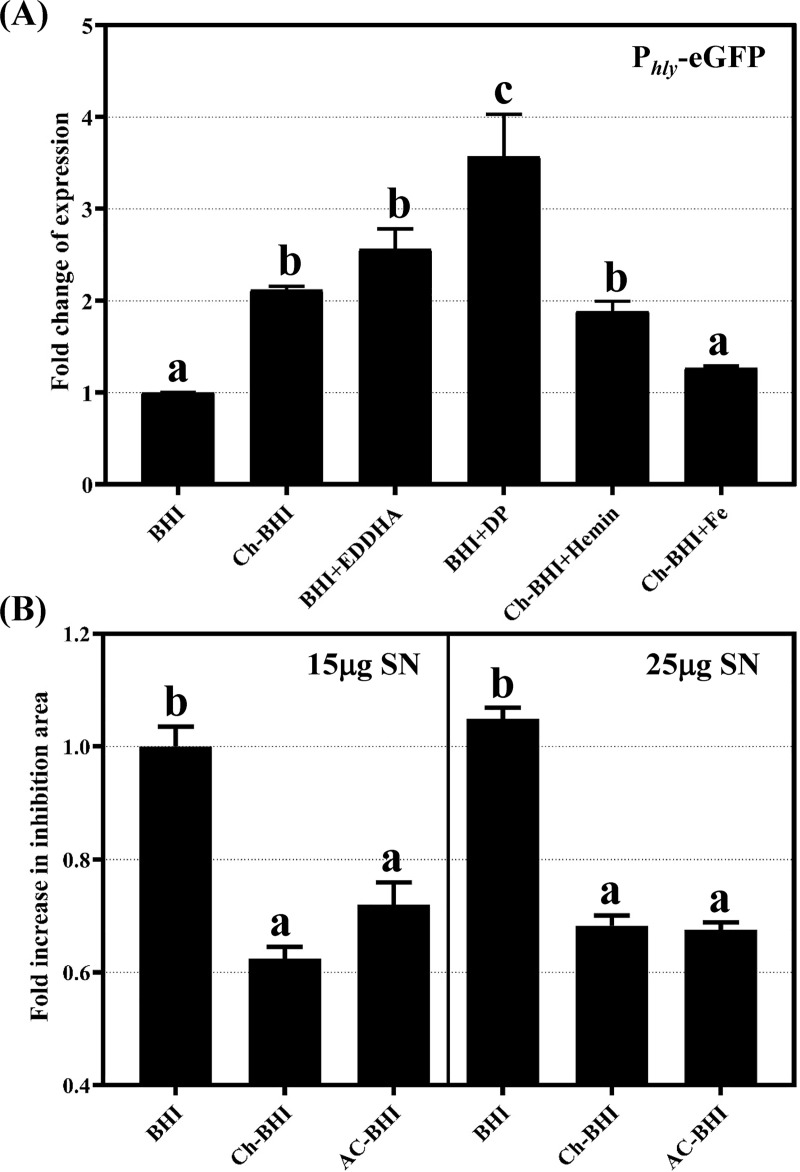
Intra- and extracellular iron depletion induces *hly* expression. (A) Effect of iron-specific chelators on expression of P_*hly*_-eGFP. An *L*. *monocytogenes* strain expressing a P_*hly*_-eGFP reporter fusion was grown in BHI or Ch-BHI in the presence of 100 μM iron, 50 μM hemin, 1mM dipyridyl (DP) or 1mM ethylenediamine-N,N′-bis(2-hydroxyphenylacetic acid) (EDDHA). GFP fluorescence levels were measured and normalized by culture OD_600_. Fold change of expression (for graphing purpose) was calculated as a ratio of normalized GFP fluorescence level relative to the normalized fluorescence level of *L*. *monocytogenes* cells expressing the reporter fusion and growing in untreated BHI. Data represent the mean of at least 3 independent biological replicates; error bars represent SEM. Bars that share the same letter are not significantly different (*p-*value > 0.05) as determined by one-way ANOVA and Tukey’s post hoc test (B) Streptonigrin susceptibility of *L*. *monocytogenes* grown on BHI, Ch-BHI or AC-BHI plates. Zones of inhibition were measured around paper disks impregnated with 15mg or 25mg streptonigrin. Fold change in inhibition area was calculated in reference to inhibition area of a strain grown on BHI. Data represent the mean of at least 3 independent biological replicates; error bars represent SEM. Bars that share the same letter are not significantly different (*p-*value > 0.05) as determined by one-way ANOVA and Tukey’s post hoc test.

Chelex and activated charcoal are general metal-chelating resins that bind different metal ions present in the growth medium with variable affinities [50,51]. Hence, we examined the effects of specific intra- and extracellular iron chelators on P_*hly*_*-*eGFP expression (Fig 3A). Ethylenediamine-N,N′-bis(2-hydroxyphenylacetic acid) (EDDHA), and the cell-permeable dipyridyl (DP) are strong Fe^3+^- and Fe^2+^-specific chelators, respectively, that are commonly used to induce iron starvation in bacteria [52,53]. In BHI, *hly* expression was induced 2.5- and 3.5 fold in the presence of EDDHA and DP, respectively, as supported by significantly higher fluorescence levels in *L*. *monocytogenes* cells expressing P_*hly*_-eGFP and grown in BHI supplemented with EDDHA and DP relative to BHI-grown cells (Fig 3A). P_*hly*_*-*eGFP expression in the presence of DP was significantly higher compared to EDDHA, which suggests that specific chelation of iron and especially the depletion of the intracellular Fe^2+^ pool effectively induces P_*hly*_*-*eGFP expression. Further, the addition of a simple iron source (FeCl_3_), but not a complex iron source (hemin), negated the effect of BHI Chelex treatment on P_*hly*_*-*eGFP expression.

### Cytosolic labile iron level is reduced in cells grown in AC-BHI and Ch-BHI

As we showed that exogenous iron repressed *hly* expression in Chelex-treated BHI (Fig 2A), we hypothesized that activated charcoal may also reduce the labile iron pool in *L*. *monocytogenes* cytosol; this is not a given as bacteria strictly regulate metal homeostasis [54] and hence levels of metal ion in the growth medium might not accurately reflect the levels of intracellular bioavailable metal ion. While atomic absorption and inductively coupled plasma mass spectrometry have previously been used to measure the intracellular metal pool, this approach quantifies both the sequestered metal ions, which are biologically unavailable, and the labile metal ion pool, which is biologically available [55]. On the other hand, streptonigrin is an antibiotic that is activated only in the presence of intracellular free iron and its effectiveness is correlated to bioavailable iron levels [56]. Hence, we used streptonigrin to estimate the intracellular labile pool of iron; this approach has previously been used in different bacteria including in *L*. *monocytogenes* [57]. To assess the levels of free intracellular iron pools, we specifically tested streptonigrin susceptibility of *L*. *monocytogenes* grown on BHI, AC-BHI or Ch-BHI plates (Fig 3B). The reduced susceptibility of *L*. *monocytogenes* cells grown on Ch-BHI or AC-BHI relative to that of cells grown on BHI indicates lower intracellular iron levels in the cells growing on Ch-BHI or AC-BHI (Fig 3B). Moreover, it is apparent that *L*. *monocytogenes* cells grown in the presence of activated charcoal and in Ch-treated BHI have similar levels of intracellular labile iron, as indicated by the comparable levels of streptonigrin resistance of *L*. *monocytogenes* cells grown in AC-BHI and Ch-BHI media.

Overall, our results show that increased expression of the PrfA-regulated P_*hly*_*-*eGFP in *L*. *monocytogenes* is correlated with the reduction of extracellular or intracellular iron levels, which was achieved either by using specific chelators or by treating growth media with Chelex or activated charcoal.

### Exogenous GSH induces *hly* expression in metal-depleted BHI medium

**Fig 4:**
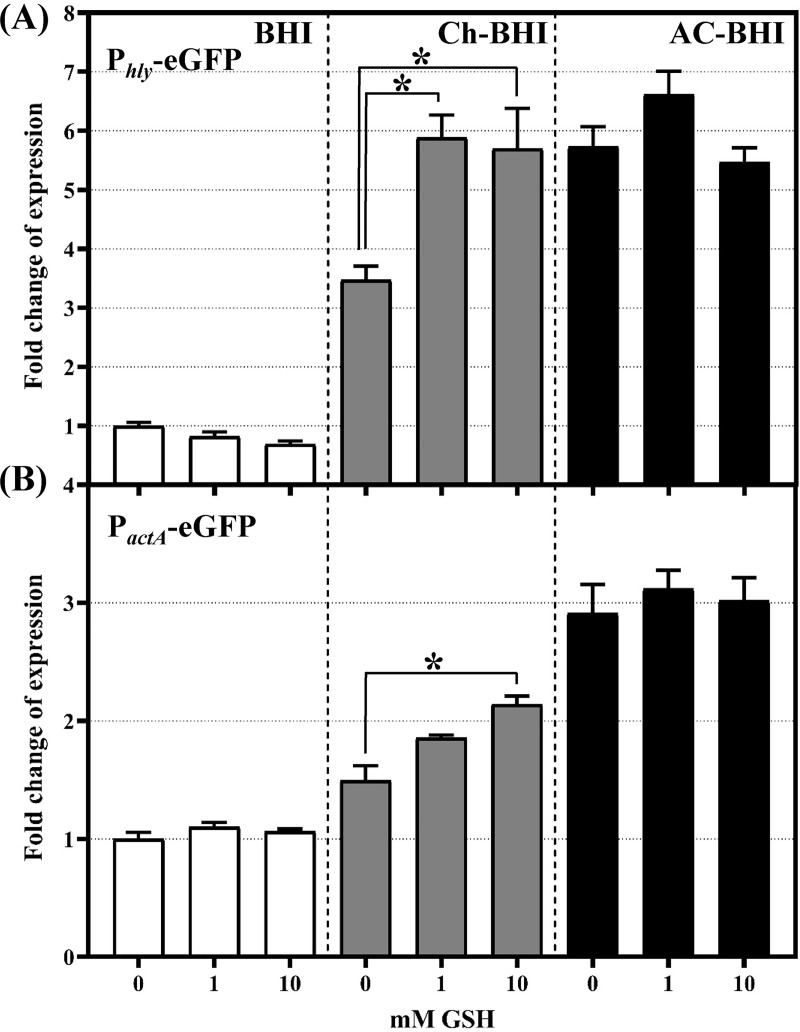
Expression of P_*hly*_-eGFP and P_*actA*_-eGFP in the presence of exogenously added glutathione (GSH). *L*. *monocytogenes* strains expressing P_*hly*_-eGFP (A) or P_*actA*_-eGFP (B) reporter fusions were grown in BHI (white bars), Ch-BHI (grey bars) or AC-BHI (black bars) with 0, 1 or 10 mM GSH. GFP fluorescence levels were measured and normalized by culture OD_600_. Fold change of expression (for graphing purpose) was calculated as a ratio of normalized GFP fluorescence level relative to the normalized fluorescence level of *L*. *monocytogenes* cells expressing the reporter fusion and growing in untreated BHI. Statistical analysis was done using the GFP/OD_600_ data from at least 3 independent biological replicates; error bars represent SEM. An asterisk denotes a significant difference between the normalized fluorescence level at a given GSH concentration and the normalized fluorescence level in the absence of GSH (*p-*value *≤* 0.05) as determined by one-way ANOVA and Tukey’s post hoc test.

Glutathione is one of the most abundant low molecular weight thiols in many organisms; GSH plays multifaceted roles in maintaining the cytosol in a reduced state, in detoxification pathways, and acts as antioxidant [58]. Moreover, GSH is a key component for buffering labile metal ions, especially iron and zinc, in both bacteria [59] and in mammalian cell cytosol and intestinal lumen [60,61], hence protecting against metal ion toxicity [59,62]. It has been widely shown that exogenously added GSH activates PrfA-dependent gene expression in chemically defined media but not in rich media [18,23,26,63]. Indeed, concentrations up to 10 mM GSH did not induce P_*hly*_*-*eGFP or P_*actA*_*-*eGFP expression in *L*. *monocytogenes* cells growing in BHI (Fig 4A). On the other hand, the addition of GSH to Ch-BHI significantly increased P_*hly*_-eGFP expression in comparison to expression in Ch-BHI with no added GSH (Fig 4A).

While P_*hly*_*-*eGFP expression was significantly increased in metal-depleted BHI in the presence of both 1mM and 10 mM GSH (Fig 4A), P_*actA*_*-*eGFP expression was only significantly induced in the presence of 10mM GSH (Fig 4B). This is consistent with previous studies that showed that *actA* transcription requires a higher level of active PrfA than *hly* transcription [64,65]. Similar to metals, exogenously added GSH did not affect the expression of either P_*hly*_-eGFP or P_*actA*_*-*eGFP in AC-BHI (Fig 4A & 4B). However, it has been shown that activated charcoal efficiently binds GSH [66]. GSH concentrations above 10mM were not tested, since at high concentrations, GSH can provide a reducing environment, which had previously been shown to induce PrfA-dependent gene expression [23–26]. Therefore, we cannot rule out the possibility that the presence of activated charcoal during growth reduces GSH bioavailability to *L*. *monocytogenes* cells [66]. These data suggest that metal ion depletion and exogenous GSH have positive synergistic effect on expression of PrfA-regulated genes.

### Differential expression of PrfA-regulated genes

We showed that metal-depletion and GSH modulate PrfA-dependent *hly* and *actA* transcription in BHI. A plethora of published data support the notion that genes in the PrfA regulon are differentially regulated to allow expression of virulence-related functions at specific phases of infection and within specific host cell compartments (for review, see [6,7]). Hence, we hypothesized that a number of PrfA-regulated genes might respond differently to metal-depletion and exogenously added GSH.

**Fig 5:**
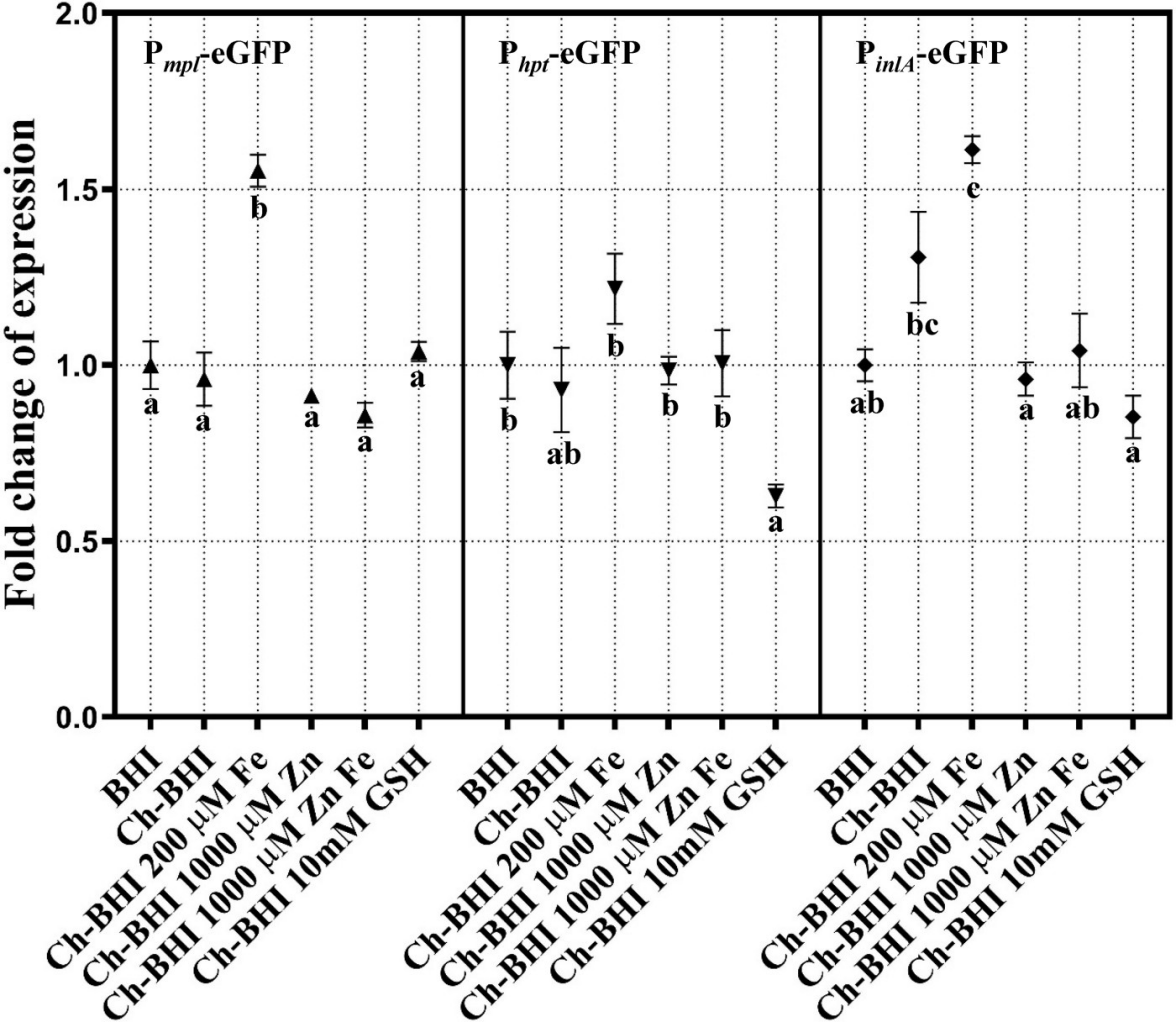
Differential expression of P_*inlA*_-eGFP, P_*hpt*_-eGFP and P_*mpl*_-eGFP. *L*. *monocytogenes* strains expressing P_*inlA*_-eGFP (♦), P_*hpt*_-eGFP (▼) and P_*mpl*_-eGFP (▲) reporter fusions were grown in BHI or Ch-BHI with or without 200 μM iron, 1,000 μM zinc or 10 mM glutathione. GFP fluorescence levels were measured and normalized by culture OD_600_. Fold change of expression (for graphing purpose) was calculated as a ratio of normalized GFP fluorescence level relative to the normalized fluorescence level of *L*. *monocytogenes* cells expressing the reporter fusion and growing in untreated BHI. Statistical analysis was done using the GFP/OD_600_ data from at least 3 independent biological replicates; error bars represent SEM. Conditions that share the same letter are not significantly different (*p-*value > 0.05) as determined by two-way ANOVA and Tukey’s post hoc test.

To further evaluate the effect of depletion of metal ion from BHI on the expression of different PrfA-regulated genes, we constructed P_*inlA*_-eGFP, P_*mpl*_-eGFP and P_*hpt*_-eGFP promoter transcriptional fusions. The native RBS in each promoter region was deleted and replaced by a canonical RBS to circumvent differences that may arise from variable translation efficiencies of eGFP transcripts in different constructs. Under these conditions, expression of P_*inlA*_-eGFP, P_*mpl*_-eGFP and P_*hpt*_-eGFP were not significantly affected by Chelex-treatment of BHI (Fig 5). Surprisingly, iron modestly induced P_*inlA*_-eGFP expression (adjusted *p*-value <0.0001 for Ch-BHI+Fe vs BHI and 0.051 for Ch-BHI+Fe vs Ch-BHI), and P_*mpl*_-eGFP expression (adjusted *p*-value <0.0001 for Ch-BHI+Fe vs Ch-BHI). On the other hand, GSH reduced the expression of P_*inlA*_-eGFP in Ch-BHI (adjusted *p*-value 0.0011 for Ch-BHI vs Ch-BHI+GSH, Fig 5). Under the tested growth conditions, P_*hpt*_-eGFP expression was not affected by Chelex treatment of BHI or by addition of iron, zinc, or GSH to Ch-BHI medium. These data indicate that relative levels of different metal ions and GSH differentially influence PrfA-dependent transcription of genes in the PrfA regulon, further supporting the notion that multiple extra- and intra-cellular cues are involved in the differential expression of members of the PrfA regulon [6,7].

## Discussion

Metals participate in many biological processes and are essential for the survival of all living organisms. One-quarter to one-third of all proteins are estimated to require metal ion [67]. In the host, metals are valuable and scarce resources for bacterial pathogens [54,68,69]; hence, metal ion homeostasis plays a major role in host-pathogen interactions and virulence [68,70]. While the host resorts to elaborate schemes to restrict metal availability (i.e., nutritional immunity), the pathogen employs counter-strategies to overcome these measures [54,68,70,71]. To illustrate, *L*. *monocytogenes* utilizes several strategies to obtain iron and zinc in intra- and extra-host environments, and such uptake systems are essential for full virulence and survival in the host (comprehensively reviewed in [69,72]). On the other end of the spectrum, the host’s innate immune system defends against bacterial pathogens by driving a rapid influx of metal ion into pathogen-containing phagosomes to cause metal-mediated-toxicity-killing [73]. Hence, bacteria strictly regulate the import and export of metal ion to maintain extremely low levels of free metal ion in the cytosol [67].

Consequently, metal efflux systems also are required for full virulence in several bacterial pathogens [73–75]. For example, *L*. *monocytogenes* encodes a P-type ATPase that functions as an Fe^2+^ efflux system critical for virulence that is required to prevent iron intoxication [76]. Despite the importance of metal iron in bacterial survival in the host and despite strong evidence that expression and activity of PrfA are modulated by multiple environmental, host and endogenous cues [7,18,21,25,26,77,78], there is surprisingly limited information on the link between metal availability and PrfA activity. In this study, we demonstrated that abundance of metal ions in complex medium plays a role in the expression of PrfA-regulated genes.

### Relative levels of metal ions influence the expression of PrfA-regulated genes

Metal content of bacteriological media varies by supplier [79]; metal content in a complex medium such as BHI broth has been reported to range from 5 μM to 15 μM for iron, 6 μM to 26 μM for zinc, and 75nM to 1.1 μM for manganese [39,80,81]. Depending on the growth medium pH and the treatment time, Chelex can reduce zinc and iron concentrations by more than 95% [82,83]. Consistent with these findings, it has been shown that pre-treatment of growth media with Chelex induces metal starvation in *L*. *monocytogenes* and other bacterial species [39,53,79]. Our data suggest that metal ions contribute to the low expression of PrfA-regulated genes in complex media, such as BHI. Indeed, both metal depletion from BHI and chelation of intracellular iron induced P_*hly*_-eGFP expression. This was corroborated by the fact that the addition of iron and zinc to Chelex-treated BHI restored the lower expression levels of *hly* and *actA*. The presence of activated charcoal throughout the course of bacterial growth, which is essential for PrfA activation [36,37], will likely results in the chelation of exogenously added metal ion and GSH, thus reducing their bioavailability to *L*. *monocytogenes* cells. Activated charcoal is a complex resin that binds multiple organic and inorganic molecules. Thus, the marginal reduction of P_*hly*_*-*eGFP expression in AC-BHI medium supplemented with zinc, but not iron and zinc, is probably due to the complex nature of activated charcoal binding to multiple compounds. Hence, a direct role of activated charcoal-dependent metal chelation in the activation of PrfA in BHI medium could not be verified. However, our data show that *L*. *monocytogenes* cells growing in AC-BHI medium have lower intracellular iron levels in comparison to cells growing in BHI medium (Fig 3B). We surmise that it is likely that activated charcoal induces PrfA-dependent expression in BHI medium by chelation of multiple molecules, which may include metal ion and oligopeptides. This is supported by previous observations that Amberlite, a polymeric non-polar resin that chelates small organic hydrophobic compounds [49,84], but lacks ion-exchange capabilities and does not bind polar compounds [37,84] also induces PrfA activity in both Amberlite treated media [23] and in media with Amberlite present [27–29]. Consequently, Amberlite XAD-4 is a poor metal-chelating resin [85], while activated charcoal is able to chelate both organic non-polar compounds and heavy metals [50,51,86].

### Differential expression of PrfA-regulated genes

To infect a host, a pathogen is compelled to express not only a multitude of proteins, regulatory RNAs and metabolites but also to fine-tune expression of different regulatory circuits. *L*. *monocytogenes* senses host cell compartments and responds by differentially regulating expression of the “right” function at the “right” time ([28–30,32,87]; for reviews see [6,7]). For example, *L*. *monocytogenes* expresses genes required for phagosome escape (e.g., *hly* and *plA*) within the host vacuole, while genes required for survival and cell-to-cell spread (e.g., *actA*, *hpt* and *inlC*) are expressed in host cytosol [28,32,33,88]. Transcription of PrfA-regulated genes appear to rely on at least three different factors: i) PrfA protein levels as influenced by *prfA* transcriptional regulation and protein half-life; ii) PrfA activity as influenced by binding to GSH for activation or oligopeptides for inhibition; and iii) conservation of the PrfA box in the promoter region of a PrfA-regulated gene.

While host GSH plays a major role in virulence in many bacterial pathogens [89], host GSH is not able to solely induce expression of *actA* within macrophages [18]. However, several lines of evidence suggest that both metal abundance and GSH bioavailability might influence the differential expression of the PrfA regulon. Interestingly, prior to the identification of GSH as a PrfA activator [18], a cell-free lysate of *L*. *monocytogenes* was shown to contain a factor that enhanced PrfA binding to its target promoter [90]. The ability of this factor to enhance PrfA binding was strongly inhibited by iron for the *actA* and *hly* promoter regions, but was enhanced for binding to the *inlA* promoter region [90,91], consistent with our findings reported here. Excess iron has also been previously shown to enhance *L*. *monocytogenes* invasion of Caco-2 cells and to be correlated with a higher-level of expression for *inlAB* [52]. On the other hand, iron depletion enhanced actin polymerization and induced expression of *actA* in the same cell line [92]. In addition, a Y154C *prfA* mutant strain was shown to invade a host cell and escape the phagosome, but was unable to induce actin polymerization and cell-to-cell spread [93], a phenocopy to a GSH-null strain [65]. A PrfA-GSH structure revealed that Y154 is needed to form a hydrogen bond with the glycine end of GSH [94,95]. PrfA activates *in vitro* transcription of promoters with perfect PrfA-boxes, such as P*plcA*, in the absence of GSH while the *actA* promoter, with an imperfect PrfA box, is not transcribed under the same conditions [96]. Our analysis of expression from *hly*, *actA*, *mpl*, *hpt* and *inlA* promoters in response to metal depletion, zinc and iron excess and addition of exogenous GSH indicates that metal abundance and exogenous GSH differentially affect expression of these promoters. Our analyses specifically showed that addition of iron to a growth medium negatively affected *hly* and *actA* expression while enhancing *inlA* expression. These data suggest that GSH bioavailability levels and metal abundance may specifically and differentially affect transcription of a sub-set of the PrfA-regulon, even though it remains unclear if variation in metal ion concentrations in different host compartments may influence the expression of PrfA-regulated genes in *L*. *monocytogenes*. While transcriptional reporters are a powerful tool to assess gene expression, several factors might influence reporter protein expression pattern and level, such as the cloning strategy, sequence variation of the RBSs, and reporter protein stability. Hence, other alternative approaches, such as quantitative RT-PCR, will be needed to validate our findings. Moreover, global approaches, such as RNA-seq, could be valuable for validation, as they would assess genome-wide changes in gene expression induced by metal ion depletion; comparative RNA-seq data on *L*. *monocytogenes* in metal ion depleted media and in infected tissue culture cells may also yield data in the *in vivo* relevance of metal ion mediated *prfA* transcription.

## Conclusions

In this study, we show that different levels of iron and zinc concentrations affect PrfA-regulated virulence gene expression. These findings further elucidate the complex interactions that contribute to the regulation of PrfA expression and activity in different environments and cellular compartments. Continued refinement of our understanding of factors that influence the ability of this pathogen to express virulence genes ultimately may lead to the development of more effective strategies for reducing the public health burden of this foodborne pathogen.

## Supporting information

S1 Fig Expression of P_*hly*_-eGFP and P_*actA*_-eGFP in the presence of iron, zinc or both ion in AC-BHI medium. *L*. *monocytogenes* strains expressing P_*hly*_-eGFP (A) or P_*actA*_-eGFP (B) reporter fusions were grown in BHI and in AC-BHI, supplemented iron, zinc or both. GFP fluorescence levels were measured and normalized by culture OD_600_. Fold change of expression (for graphing purpose) was calculated as a ratio of normalized GFP fluorescence level relative to the normalized fluorescence level of *L*. *monocytogenes* cells expressing the reporter fusion and growing in untreated BHI. Statistical analysis was done using the GFP/OD_600_ data from at least 6 independent biological replicates; error bars represent SEM. Asterisks denote significant difference between the normalized fluorescence level at the given condition compared to normalized fluorescence level in AC-BHI (*p-*value *≤* 0.05,) as determined by one-way ANOVA and Dunnett’s post hoc test.
(TIF)

S2 Fig Expression of P_*hly*_-eGFP in the presence of iron, zinc or both. *L*. *monocytogenes* strain expressing a P_*hly*_-eGFP reporter fusion was grown in BHI supplemented with different concentrations of iron, zinc, or both. GFP fluorescence levels were normalized by culture OD_600_. Fold change of expression (for graphing purpose) was calculated as a ratio of normalized GFP fluorescence level relative to the normalized fluorescence level of *L*. *monocytogenes* cells expressing the reporter fusion and growing in untreated BHI. Data represent the mean of at least 3 independent biological replicates; error bars represent SEM.
(TIF)

S1 Table Strains used in this study. (PDF)

S2 Table Oligonucleotides used in this study. (PDF)
