# Supplementary figures and images for: Characterization of the roles of activated charcoal and Chelex in the induction of PrfA regulon expression in complex medium
Source: PLoS One. 2021 Apr 29;16(4):e0250989. doi: 10.1371/journal.pone.0250989 (PMC8084165; doi:10.1371/journal.pone.0250989)

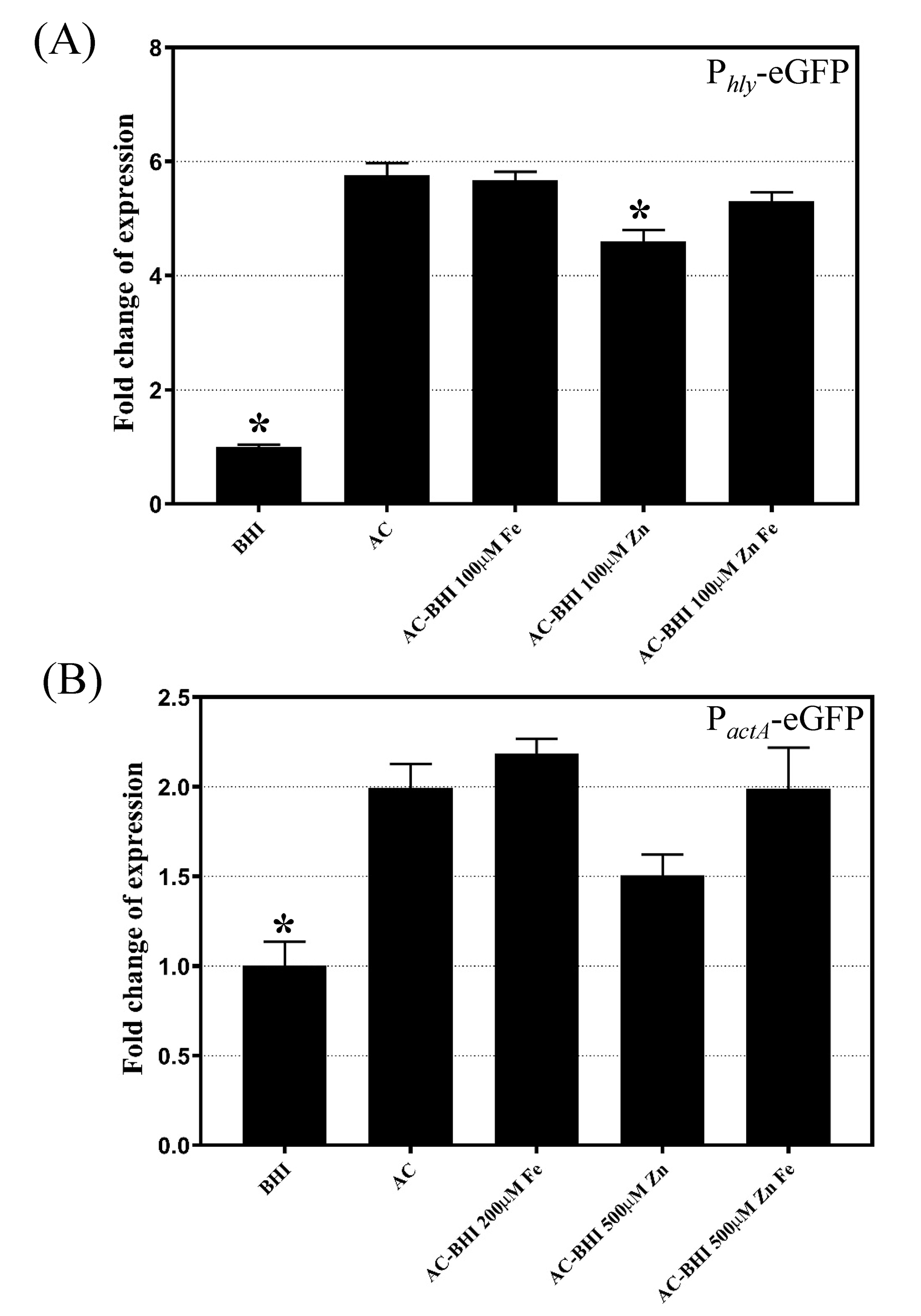

Supplement: S1 Fig — L. monocytogenes strains expressing Phly-eGFP (A) or PactA-eGFP (B) reporter fusions were grown in BHI and in AC-BHI, supplemented iron, zinc or both. GFP fluorescence levels were measured and normalized by culture OD600. Fold change of expression (for graphing purpose) was calculated as a ratio of normalized GFP fluorescence level relative to the normalized fluorescence level of L. monocytogenes cells expressing the reporter fusion and growing in untreated BHI. Statistical analysis was done using the GFP/OD600 data from at least 6 independent biological replicates; error bars represent SEM. Asterisks denote significant difference between the normalized fluorescence level at the given condition compared to normalized fluorescence level in AC-BHI (p-value ≤ 0.05,) as determined by one-way ANOVA and Dunnett’s post hoc test. (TIF) [file pone.0250989.s001.tif]

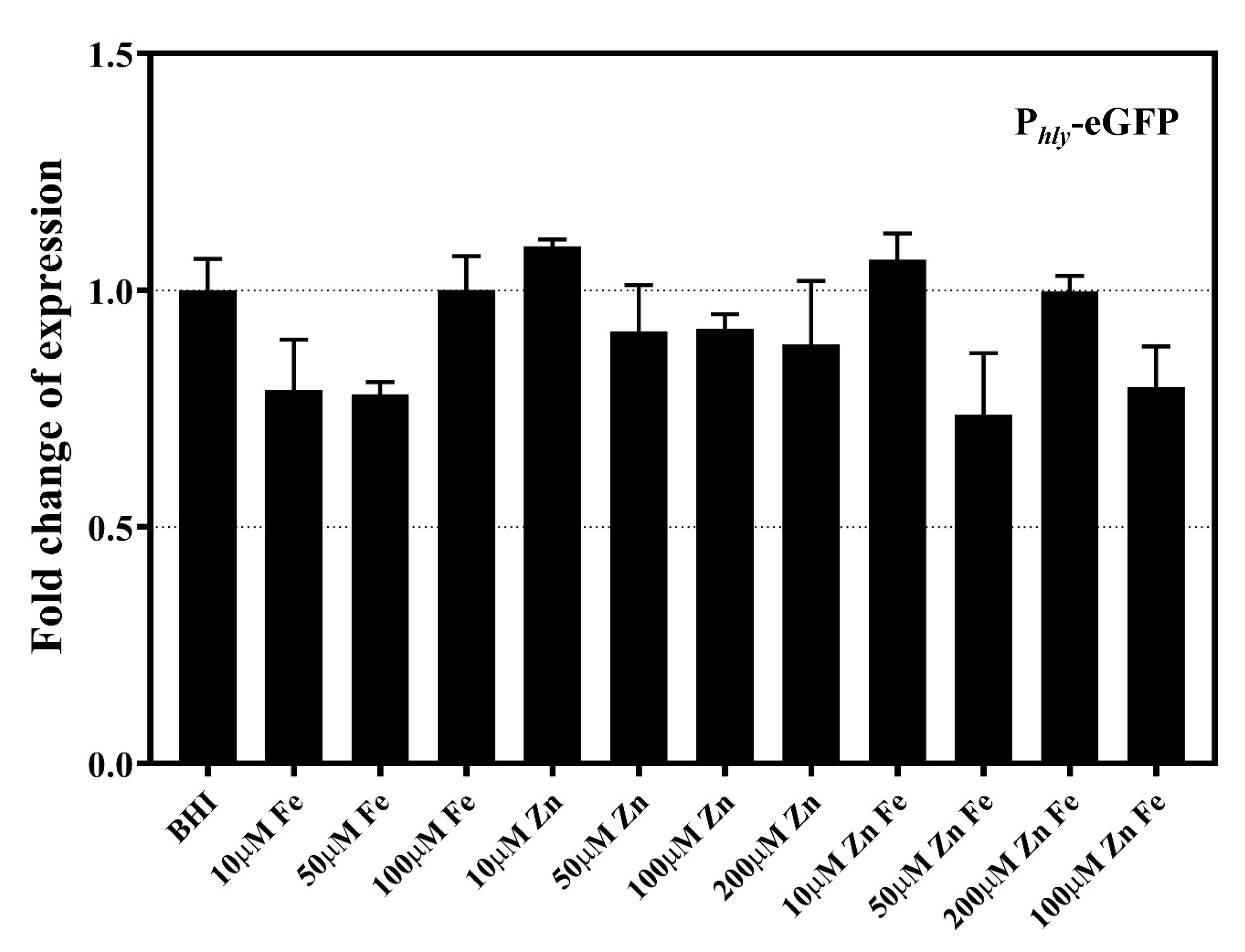

Supplement: S2 Fig — L. monocytogenes strain expressing a Phly-eGFP reporter fusion was grown in BHI supplemented with different concentrations of iron, zinc, or both. GFP fluorescence levels were normalized by culture OD600. Fold change of expression (for graphing purpose) was calculated as a ratio of normalized GFP fluorescence level relative to the normalized fluorescence level of L. monocytogenes cells expressing the reporter fusion and growing in untreated BHI. Data represent the mean of at least 3 independent biological replicates; error bars represent SEM. (TIF) [file pone.0250989.s002.tif]
