# Supplementary material for: Characterization of the roles of activated charcoal and Chelex in the induction of PrfA regulon expression in complex medium
Source: PLoS One. 2021 Apr 29;16(4):e0250989. doi: 10.1371/journal.pone.0250989 (PMC8084165; doi:10.1371/journal.pone.0250989)
Supplement: S1 Table — (PDF) [file pone.0250989.s003.pdf]

Strain	Strain/relevant genotype	Source
E. coli	NEB5α / *fhuA2* Δ*(argF-lacZ)U169 phoA glnV44* Φ*80* Δ*(lacZ)M15 gyrA96 recA1 relA1 endA1 thi-1 hsdR17*	New England Biolabs
E. coli	SM10 λpir / F^{-}, *thi-1, thr-1, leuB6, recA, tonA21, lacY1, supE44,* Mu, λ^{-}, [RP4-2(TC::Mu)] Km^{R}, Tra^{+}	[1]
E. coli	COB631 pKSV7-$P_{lmo2230}$::*egfp*	[4]
E. coli	FSL G4-0081 SM10 pPL2 containing P_{inlA}::eGFP	This study
E. coli	FSL G4-0082 SM10 pPL2 containing P_{hly}::eGFP	This study
E. coli	FSL G4-0083 SM10 pPL2 containing P_{hpt}::eGFP	This study
E. coli	FSL G4-0084 SM10 pPL2 containing P_{mpl}::eGFP	This study
E. coli	FSL G4-0085 SM10 pPL2 containing P_{actA}::eGFP	This study
Listeria monocytogenes	10403S Wild type	[2]
Listeria monocytogenes	FSL B2-0237 *prfA**(G155S)	[3]
L. monocytogenes	FSL G4-0042 10403S $tRNA^{Arg}$:: pPL2-P_{hly}::eGFP	This study
L. monocytogenes	FSL G4-0045 10403S $tRNA^{Arg}$:: pPL2- P_{actA}::eGFP	This study
L. monocytogenes	FSL G4-0048 10403S $tRNA^{Arg}$:: pPL2- P_{mpl}::eGFP	This study
L. monocytogenes	FSL G4-0051 10403S $tRNA^{Arg}$:: pPL2- P_{hpt}::eGFP	This study
L. monocytogenes	FSL G4-0054 10403S $tRNA^{Arg}$:: pPL2- P_{inlA}::eGFP	This study
L. monocytogenes	FSL G4-0043 *prfA** $tRNA^{Arg}$:: pPL2-P_{hly}::eGFP	This study

References.

1.Simon R, Priefer U, Pühler A. A Broad Host Range Mobilization System for In Vivo Genetic Engineering: Transposon Mutagenesis in Gram Negative Bacteria. Bio/Technology. 1983;1(9):784-91. doi: 10.1038/nbt1183-784.

2.Bishop DK, Hinrichs DJ. Adoptive transfer of immunity to *Listeria monocytogenes*. The influence of *in vitro* stimulation on lymphocyte subset requirements. Journal of immunology (Baltimore, Md : 1950). 1987;139(6):2005-9. Epub 1987/09/15. PubMed PMID: 3114382.

3.Ollinger J, Bowen B, Wiedmann M, Boor KJ, Bergholz TM. *Listeria monocytogenes* sigmaB modulates PrfA-mediated virulence factor expression. Infect Immun. 2009;77(5):2113-24. Epub 2009/03/04. doi: 10.1128/IAI.01205-08. PubMed PMID: 19255187; PubMed Central PMCID: PMCPMC2681731.

4.Utratna M, Cosgrave E, Baustian C, Ceredig R, O'Byrne C. Development and optimization of an EGFP-based reporter for measuring the general stress response in *Listeria monocytogenes*. Bioeng Bugs. 2012;3(2):93-103. Epub 2012/04/28. doi: 10.4161/bbug.19476. PubMed PMID: 22539028; PubMed Central PMCID: PMCPMC3357339.
