# Supplementary material for: Characterization of the roles of activated charcoal and Chelex in the induction of PrfA regulon expression in complex medium
Source: PLoS One. 2021 Apr 29;16(4):e0250989. doi: 10.1371/journal.pone.0250989 (PMC8084165; doi:10.1371/journal.pone.0250989)
Supplement: S2 Table — (PDF) [file pone.0250989.s004.pdf]

Primer name	Sequence	Purpose
AG67-115	ATACTGCAGTAAGGAGGTTGGAATATGGTTAGTAAAGGAGAGGAAT	PCR amplification of *eGFP* from pKSV7-$P_{lmo2230}$::*egfp* [4]
AG67-116	ATAGAGCTCTTATTTGTATAATTCATCCATTCCT	
AG67-97	GAACAAAAGCTGGTACCGGGTCCTATCTTAAAGTTACTTTTATG	PCR amplification of P_{hly} from *L. monocytogenes*
AG67-98	AGATCCCCCGGGCTGCAGGAACTACATTTTTTAACCTAATAATGCC	
AG67-99	GAACAAAAGCTGGTACCGGGTGATAAAAAATTAATGTAAGATAAGT	PCR amplification of P_{inlA} from *L. monocytogenes*
AG67-100	AGATCCCCCGGGCTGCAGGAAAATAGTTAGAAACAATATCAGGTT	
AG67-101	GAACAAAAGCTGGTACCGGGAAGATTTATATGGTGAAGATGCT	PCR amplification of P_{actA} from *L. monocytogenes*
AG67-102	AGATCCCCCGGGCTGCAGGAACCTCGTGATACGCTAATACAA	
AG67-105	AGTGGTACCTAAAACCGCTTAACACACACGA	PCR amplification of P_{mpl} from *L. monocytogenes*
AG67-106	AGACTGCAGAACTAAGTTTAAGCCACCTACA	
AG67-107	ATAGGTACCGGTTTCAGGGAATGTTTTACTA	PCR amplification of P_{hpt} from *L. monocytogenes*
AG67-108	ATACTGCAGCAGTGTAATCTGCATTCTAAATT	
